# OCT-based label-free *in vivo* lymphangiography within human skin and areola

**DOI:** 10.1038/srep21122

**Published:** 2016-02-19

**Authors:** Utku Baran, Wan Qin, Xiaoli Qi, Goknur Kalkan, Ruikang K. Wang

**Affiliations:** 1 Department of Bioengineering, University of Washington, Seattle, WA, USA; 2 Department of Electrical Engineering, University of Washington, Seattle, WA, USA; 3 Department of Dermatology, Yıldırım Beyazıt University, Ankara, Turkey

## Abstract

Due to the limitations of current imaging techniques, visualization of lymphatic capillaries within tissue *in vivo* has been challenging. Here, we present a label-free high resolution optical coherence tomography (OCT) based lymphangiography (OLAG) within human skin *in vivo*. OLAG enables rapid (~seconds) mapping of lymphatic networks, along with blood vessel networks, over 8 mm x 8 mm of human skin and 5 mm x 5 mm of human areola. Moreover, lymphatic system’s response to inflammation within human skin is monitored throughout an acne lesion development over 7 days. The demonstrated results promise OLAG as a revolutionary tool in the clinical research and treatment of patients with pathologic conditions such as cancer, diabetes, and autoimmune diseases.

Lymphatic system drains lymph fluid from the extracellular space into larger ducts through unidirectional, thin-walled capillaries and collecting vessel network. To transport the fluid, lymphangions, the vascular smooth muscle cells, rhythmically constrict and relax, and valve leaflets open and close in an orchestrated manner to mediate unidirectional flow^1^. This mechanism is critical for tissue fluid homeostasis and immune cell transport and its dysfunction causes symptoms of edema^2^. Early detection of dysfunctional lymphatic transport in asymptomatic patients before the onset of symptoms could enable earlier diagnoses and more effective treatments. In addition, the identification of factors that affect lymphatic pumping is an active area of research that will contribute to the development of new pharmacologic strategies to correct lymphatic insufficiency.

Most of the current and emerging lymphatic imaging approaches require the administration of exogenous contrast agents either directly into lymphatics via the cannulation of a lymphatic vessel or indirectly into lymphatic plexus via intradermal injection. Unfortunately, most of the existing contrast agents can be toxic and induce side effects to patients^3^. Moreover, locating and cannulating a lymphatic vessel can be significantly invasive for patients and very difficult in preclinical research using transgenic mouse models due to their small size^4^. X-ray^5^, magnetic resonance imaging^6^, near infrared fluorescence imaging^7^ and lymphoscintigraphy^8^ techniques provide a body imaging, albeit with poor resolution, so they are used for imaging the larger vessels and the lymph nodes. On the other hand, a microscope based imaging technique, fluorescence microlymphography^9^, is limited with its penetrating depth (~200 μm) in tissue but can provide relatively higher resolution (~50 μm), hence it is used only in the visualization of initial lymphatics near injection site.

Despite its prominent role in healthy and pathological processes, there are only few methods reported in the literature^10^,^11^,^12^ for the purpose of non-invasively imaging the response of lymphatics within *in vivo* animal models of disease. So far, none of these techniques has shown its potential in clinical investigations. Optical coherence tomography (OCT) is an emerging tool for non-invasive, label-free *in vivo* imaging of tissue^13^. It can provide a better penetration depth in tissue than a microscope (~up to 2 mm) with a high resolution (~10 μm). Optical microangiography (OMAG) is a label-free non-invasive imaging and processing method to obtain 3D *in vivo* blood perfusion map in tissue beds down to capillary level using Fourier domain OCT^14^,^15^. OMAG has been applied to visualize microvasculature in various living tissue including healthy^16^,^17^ and pathological human skin^18^, and mouse cerebral cortex^19^,^20^.

Previously, label-free imaging of lymphatic vessels has been demonstrated on an *in vivo* mouse ear model using OCT^11^,^12^,^21^. The difference in optical scattering between lymph fluid, which is nearly transparent, and tissue, which is highly scattering is used as a contrast mechanism. Consequently, the lymphatic vessels appear as low-scattering (darker) regions in the OCT images. Three-dimensional connectivity between vessels and characteristic lymphangion morphology are used for the identification of lymphatic network. The result of lymphangiography maps were confirmed by intra-dermal injection of Evan’s blue dye and monitoring the uptake pathway by surrounding lymph vessels into the lymph nodes^11^,^12^. The lymphatic vessels have been visualized by applying a threshold to the OCT intensity images^21^, or using vesselness models based on Hessian multi-scale filters^12^. These methods work well on *in vivo* mouse ear models, however, they have not been able to apply to human skin due to obvious challenges facing optical imaging, i.e., the thicker and higher scattering nature of human skin compared to mouse ear skin. The contrast of the dark areas to the surrounding tissue becomes worse and segmentation algorithms fail to provide a clear separation of these areas from the surrounding tissue in OCT images of human skin.

We developed a method to contrast lymphatic vessels within highly scattering tissue by reducing light attenuation effect on OCT cross-sectional images and extracting lymphatic vessels through re-arranged enface OCT images (Supplementary Data). With this method, we are able to non-invasively visualize lymphatic networks along with microvasculature within human skin and areola, without using contrast agents. To our knowledge, this is the first demonstration of OCT based label-free optical lymphangiography (OLAG) within human skin and areola *in vivo*. Moreover, lymphatic system’s response to inflammation is monitored on an acne lesion within human skin for 7 days. Results showed that OLAG is promising to provide a remarkable alternative tool in the investigation and treatment of pathologic mechanisms involving lymphatic system without the use of exogenous contrast agents on patients.

## Results

### *In vivo* lymphangiography within human arm skin

**Figure 1:**
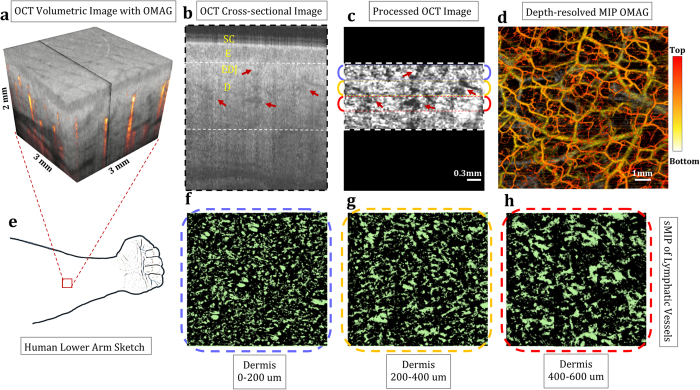
En face mapping of lymphatic vessels at different layers of human lower arm skin with 8 mm × 8 mm total imaging area. (**a**) OCT volumetric structural image overlaid with volumetric OMAG acquired within *in vivo* human lower arm skin. (**b**) OCT cross-sectional image taken from the OCT volumetric data, pointed with black line in (**a**). SC: Stratum Corneum, E: Epidermis, EDJ: Epidermal-dermal junction, D: Dermis. (**c**) Segmented and processed cross section of dermis, pointed out at (**b**) with white dashed lines. (**d**) En face OMAG images with depth-resolved MIP. (**e**) Sketch of human lower arm where OCT images are taken from. (**f–h**) En face sMIP of lymphatic vessels from 0–200 um of dermis (**f**), 200–400 um of dermis (**g**), and 400–600 um of dermis (**h**). Red arrows point out some of the lymphatic vessels seen as low-scattering (dark) regions. [Figure (**e**) art by Uktu Baran].

Using OCT, we imaged the skin in the ventral side of the human lower arm, and presented the results in Fig. 1. To achieve a large field of view, the final enface image is formed by mosaicking 9 images, each with 3 mm × 3 mm area. Depth-resolved maximum intensity projection (MIP) OMAG image shows the blood vessel network within human arm skin in Fig. 1d. Figure 1c shows the cross-section of the dermis after the compensation and the segmentation. The lymphatic vessels in the corresponding portions of the dermis are presented with enface sMIP (see Supplementary Information) in Fig. 1f,g. The results shows that the lymphatic vessels are in smaller shapes in the top layers of dermis and it becomes larger in the deeper layer, which is consistent with the literature^22^. Unlike blood vessels, lymphatic vessels are loosely attached together.

### *In vivo* lymphangiography within human breast

The areolar dermis consists of highly active lymphatic plexus^23^. Subareolar lymphatic plexus contains a rich network of lymphatic capillaries that originates in the dermis of the nipple–areolar complex, and ultimately terminates in regional lymph nodes. Because of its many interlymphatic connections, the subareolar lymphatic plexus is a great region for utilizing OLAG.

**Figure 2:**
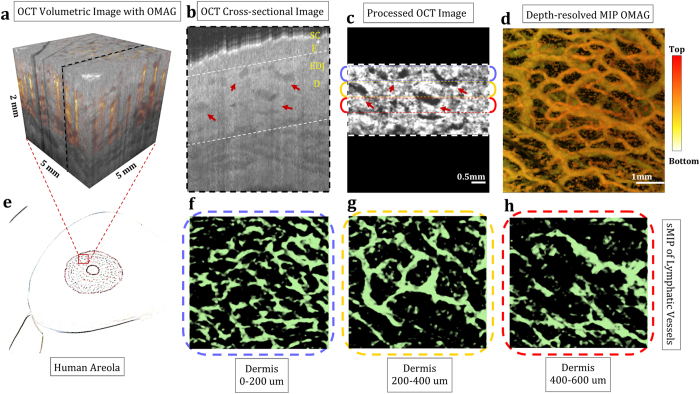
En face mapping of lymphatic vessels at different layers of human areola with 5 mm × 5 mm imaging area. (**a**) OCT volumetric structural image overlaid with volumetric OMAG acquired within *in vivo* human areola. (**b**) OCT cross-sectional image taken from the OCT volumetric data, pointed with dashed black line in (**a**). SC: Stratum Corneum, E: Epidermis, EDJ: Epidermal-dermal junction, D: Dermis. (**c**) Segmented and processed cross section of dermis, pointed out at (**b**) with white dashed lines. (**d**) En face OMAG image with depth-resolved MIP. (**e**) Sketch of human areola where OCT image is taken from. (**f–h**) En face sMIP of lymphatic vessels from 0–200 um of dermis (**f**), 200–400 um of dermis (**g**), and 400–600 um of dermis (**h**). Red arrows point out some of the lymphatic vessels seen as low-scattering (dark) regions. [Figure (**e**) art by Uktu Baran].

Figure 2 shows the results of OMAG and OLAG for human areola with 5 mm × 5 mm imaging area. MIP and depth-resolved MIP OMAG images show the rich blood vessel network within human areola in Fig. 2c,d. Figure 2b shows the cross-section of the dermis after the compensation and the segmentation. The lymphatic vessels in the corresponding portions of the dermis are shown with enface sMIP in Fig. 2f–h. Compared to arm skin, lymphatics in areola are visualized with better interconnections. This can be attributed to smaller epidermal layer and more active lymphatic plexus in areola.

### Monitoring simultaneous response of blood and lymphatic vessels to inflammation within acne lesion on human skin

**Figure 3:**
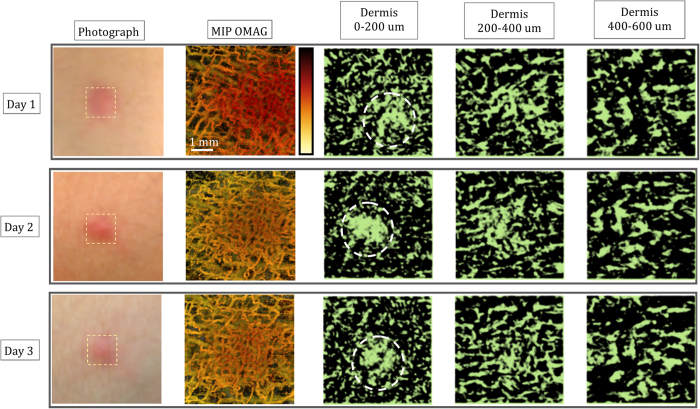
Monitoring blood and lymphatic vessels within inflamed human skin with 5 mm × 5 mm imaging area. En face depth-resolved MIP of OMAG and sMIP of OLAG images with 5 mm × 5 mm field of view are shown for day 1, 2, and 3 of acne lesion development. Blood vessels are color coded according to their position in depth and lymphatic vessels are shown in green. Imaged areas are pointed out on photographs of acne lesion. While circles are highlighting the areas with edema.

**Figure 4:**
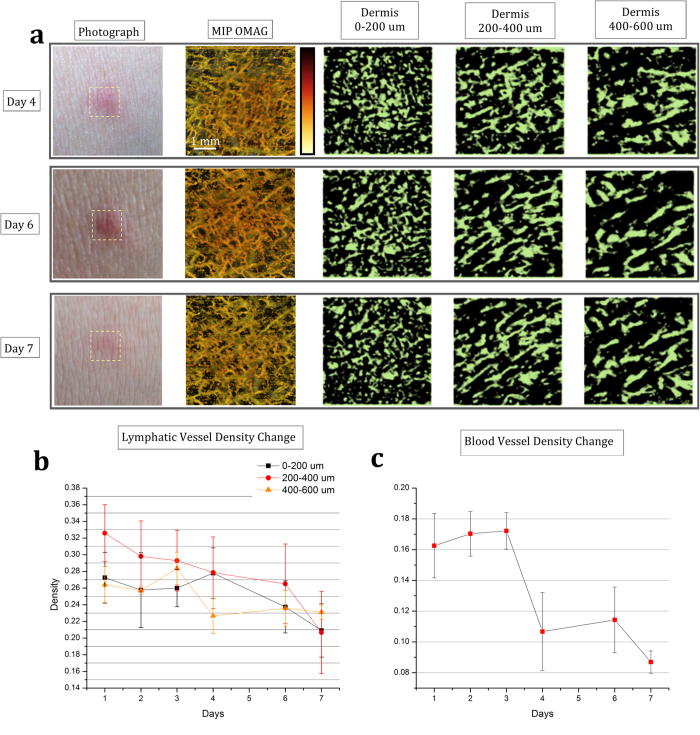
Monitoring blood and lymphatic vessels within healing human skin with 5 mm × 5 mm imaging area. (**a**) En face depth-resolved MIP of OMAG and sMIP of OLAG images with 5 mm × 5 mm field of view are shown for day 4, 6, and 7 of acne lesion development. Blood vessels are color coded according to their position in depth and lymphatic vessels are shown in green. Imaged areas are pointed out on photographs of acne lesion. (**b**) Comparison of lymphatic vessel density change observed in OLAG images for different layers over 7 days (n = 4). (**c**) Microvascular density change observed in the MIP OMAG images over 7 days (n = 4).

During inflammation, the lymphatic drainage is activated along with the vascular remodelling. Therefore, the monitoring of a simple inflammation phenomenon could be a good surrogate for demonstrating the usefulness of the proposed OLAG technique. Here, human skin’s inflammatory state and its recovery are monitored for 7 days within an acne lesion using OMAG and OLAG and shown in Figs 3 and 4. In these figures, the results for certain days are presented in each row. Accordingly, photograph, depth-resolved MIP OMAG, and the OLAG images from various depths are included in this figure. Field of view for OCT imaging is 5 mm ×5 mm.

The volumetric depth-resolved MIP of OMAG images clearly show the changes in microvascular activity within the region of interest. In the first 3 days, inflammation causes edema in the tissue, which is circled in Fig. 3. Edema region consists of inflammatory cells and water. Since there is less light scattering in this region compared to surrounding tissue, it looks darker on the OCT image, and hence appearing as an artifact in the final enface OLAG image. However, it can be differentiated from the lymphatic vessels since it is not part of the connected network. On the other hand, lymphatic vessel network is very active during the inflammatory state of acne development and lymphatic vessel density decreases as acne lesion heals, as shown in Fig. 4b. Another interesting observation is that the lymphatic vessels in the lower layers of dermis have more pronounced connections in a network compared to Fig. 1. This can be explained by the fact that lymphatic vessels are usually reserved and hard to visualize fully without activation.

Breakage of dermal balance and inflammatory response leads to dense microvasculature in Fig. 3, by activating the reserve blood vessels during the inflammatory stage of acne development^18^. With the decreased inflammation at day 4, blood and lymphatic vessel densities decrease and eventually go back to normal as shown in Fig. 4.

## Discussion

Lymphangiogenesis is critical to maintain internal stability of the immune system by reducing edema and inflammation in the tissue^24^. Several studies have pointed out the importance of lymphangiogenesis and its role in tumor metastasis in cancer progression^25^,^26^. In malignant melanomas, the presence of both intratumoral and peritumoral lymphangiogenesis was demonstrated^27^. Histological studies reveal that lymphangiogenesis may arise prior to the onset of metastasis, and an increase in lymph flow to tumor-draining lymph node has been observed^28^. It was proposed that changes in lymphatic function, remodeling and the degree of tumor lymphangiogenesis may be used to early determine the metastatic potential, lymphatic involvement and total patient survival^27^. Therefore inhibition of tumor lymphangiogenesis pharmacologically has also become an alternative promising treatment model for preventing metastasis in cancer^29^.

There are no truly non-invasive ways to assess the *in vivo* status of tumor induced lymphangiogenesis and to monitor the treatment modalities on lymphatics. Currently, subdermal injection of contrast agents is required for imaging lymphatic vessels in clinics, but they can create complications in patients so it is considered as significantly invasive in the clinical care as well as in the clinical research^4^. Injection itself might also initiate an inflammatory response which in return affects the results. In addition, it only shows a specific pathway depending on the injection site.

Here, we succeeded to visualize lymphatic vasculature within human skin and areola *in vivo*, in the absence of exogenous contrast agents. We also observed the variations in the dynamics of lymphatic vessels in the case of inflammation that has been developed by acne vulgaris. To the best of our knowledge, this is the first study imaging the lymphatic system within human skin without any interventional radiology procedures. Our technique offers a unique ability to quickly visualize the microvascular and lymphatic response to stimulation, non-invasively. It may be used to image functional and constitutional changes through lymphangiogenesis and angiogenesis in the patients with cancer in response to tumor growth. OLAG results from subareolar lymphatic plexus (Fig. 2) may offer an opportunity for early diagnosis of carcinoma without using invasive methods.

Variations in the functions of both vascular and nodal parts of the lymphatic system during inflammation processes have been described^30^. The change in the phenotype of the vessels is composed of lymphangiogenesis and lymphatic contractile dysfunction. Moreover, the increase of blood vessel remodeling, aberrant growth or activation of numerous abnormal dilated lymphatic vessels within the lesions, and the decrease of lymph flow are also key features of many chronic inflammatory skin diseases like UV damage^31^, psoriasis^32^,^33^, and bullous pemphigoid^34^. Recent studies also indicated a close association of lymphangiogenesis with the pathogenesis of rosacea^35^ and cutaneous lichen planus^36^.

In this study, we observed structural changes in the lymphatic vascular system over time during inflammation and monitored lymphatic flow changes in the skin as a result of new vessel formation (Figs 3 and 4). Since the similar changes are observed in the inflammatory disorders, these results give an opportunity to assess these diseases in the level of diagnosis, course and the response to the treatment. The grade of lymphatic vascular activation can be a biological marker for the severity and activity of these chronic inflammatory skin diseases. Therefore, we would expect that the ability of OLAG to delineate the lymphatic vessels *in vivo*, at the level of capillaries, could provide useful quantitative metrics for monitoring and assessing the consequences of treatment in these disorders.

Nevertheless, our method also comes with some limitations. Firstly, the current lateral resolution of our system was ~22 μm. This makes it harder to resolve small blood and lymphatic capillaries. However, with this resolution, this technique would be sufficient for comparison studies where errors in the measurements do not affect the differential conclusions. Secondly, hair follicles might create false positives due to their strong absorption to the OCT light source. Finally, although the accuracy of this technique has been shown (Supplementary Fig. 3) and validated on mouse models before^11^,^12^, additional studies with histopathological comparisons have to be made with human subjects to further validate and refine OLAG for human skin.

Several improvements can be made to our methods in the future. Firstly, the spatial resolution of the system needs to be improved so that smaller blood and lymphatic vessels can be imaged. However, with the increase of the system resolution, there will be penalty paid to the depth of field of the system that would ultimately render a reduced ability to see deep vessels. This issue may be mitigated, on the other hand, by the use of dynamic focus control so that the lateral resolution can be maintained throughout the imaging depth without creating an out of focus image. Secondly, in this study, we elected to image areas with few to none hair for the demonstration of proposed method. However, for haired skin, the histogram equalization method can be improved by selectively disregarding the areas with hair follicles in enface images, automatically. Finally, improving the accuracy of segmentation warrants further investigation for improved quantification of the results in various other applications aimed to understand the mechanisms how the lymphatic system responds to the disease or its treatment regimen.

In summary, we have reported an OCT based label-free optical lymphangiography for the first time within *in vivo* human skin and areola. We demonstrated the capabilities of this technique to image the microvasculature and lymphatic vessels within human skin up to capillary level without a need for exogenous contrast agents. The lymphatic circulatory system has important functions in immune surveillance and play a key role in several diseases. The currently available techniques require contrasting material being administered into human body, which can lead to complications. Label-free imaging of blood and lymphatic vessels with high resolution within human skin and areola using OMAG and OLAG may be useful in improving the patient care, and in addition, it could open up opportunities to better understand tissue response to various pathological cases such as inflammatory diseases and cancer, and consequently facilitating new treatment options targeting lymphatic system. More studies should be conducted to better understand the nature of lymphatic system’s involvement in cases such as wound healing, diabetes, and autoimmune diseases.

## Methods

### Experiment Setup

We used a swept source OCT (SS-OCT) system operating at 1310 nm central wavelength to implement our methods to image blood and lymphatic microcirculation within *in vivo* human skin (Supplementary Fig. 2). The system employed a swept light source containing a MEMS-tunable vertical cavity surface-emitting laser (VCSEL) (Thorlabs Inc.). This VCSEL source is able to sweep the wavelength of the laser across a broad spectral range near 1310 nm at a fixed repetition rate of 100 kHz, giving a long coherence length (>50 mm) with ~15 μm axial resolution in tissue and 2 mm imaging range in human skin. The sample arm consists of a probe imaging head (including galvanometric scanners) and a 5× objective lens (LSM03, Thorlabs Inc.), giving 22 μm lateral resolution.

In this study, we elected the use of SS-OCT over SD-OCT system for the following reasons: its longer imaging range, less depth-dependent signal roll-off, and less motion-induced signal loss due to fringe washout^37^. Therefore, our SS-OCT system provides a significant advantage for lymphatic imaging application within highly scattering *in vivo* human skin.

The optical power of incident light upon the sample was ~5.2 mW, well below the American National Standards Institute (ANSI) standards (Z136.1) for the safe use of near infrared light at 1310 nm on skin^38^. For animal experiments described in this paper, the procedures were approved by the Institute of Animal Care and Use Committee (IACUC) of the University of Washington and conducted in accordance with the approved protocol. For human imaging, subject volunteers were used in this study to demonstrate the usefulness of OLAG in normal skin and skins with pathological conditions. In order to mitigate the strong specular reflection from the skin surface, a mineral oil was topically applied to the skin surface. The use of OCT/OMAG laboratory instrumentations on volunteer subjects was reviewed and approved by the Institutional Review Board of the University of Washington, and informed consent was obtained from all subjects before imaging. This pilot study followed the tenets of the Declaration of Helsinki and was conducted in compliance with the Health Insurance Portability and Accountability Act.

### Optical Microangiography

To visualize the volumetric microvasculature up to capillary level, an OMAG based OCT angiography technique was utilized^15^. Briefly in this scanning protocol, volumetric OCT dataset was acquired on 5 mm × 5 mm field of view using a raster scanning provided by the galvanometric scanners. Each B-frame contained 256 A-lines. In the slow C-scan, total of 2048 B-frames at 256 spatial locations were captured with 8 repetitions at the each location, which took ~20 s with a 100 frames/s imaging rate. This scanning protocol translated to ~20 μm spacing between pixels in both x and y directions. A cross-correlation-based image registration method was applied for the adjacent B-frames in the 3D amplitude data set to compensate axial displacement induced by possible tissue bulk motion^39^. Then, OMAG algorithm^15^ based on full complex OCT signal was applied to 8 repeated B-frames at the same location to obtain a cross-sectional blood flow intensity image. This technique takes advantage of the dynamic speckle effect due to moving RBCs within functional vessel in contrast to the static OCT signal from non-flow regions (bulk tissue) or lymphatic vessel regions. By calculating the signal difference across the pixels, blood vessel morphology is visualized. Contrast is achieved since flow regions exhibit high signal fluctuation (i.e. dynamic speckle) while other areas give relatively constant values.

Acquired 3D OMAG datasets were visualized through volume rendering and a Gaussian filter of 3 × 3 kernel size was applied for noise reduction. Maximum intensity projection (MIP) of the OMAG blood flow image was produced by mapping the maximal value in each A-line on to an enface 2D plane which was used to visualize the blood vessel networks.

### OCT based Lymphangiography

Accurate detection of lymphatic vessels within human skin requires OCT images with good contrast. Non-uniform intensity distribution of the OCT images due to light attenuation and heterogeneous tissue properties make it difficult to extract lymphatic vessels within highly scattering tissue. In order to mitigate this problem, OCT structural images were processed in axial and lateral directions and the contrast was enhanced using a processing protocol described below.

The contrast of the original OCT structural images were first improved using attenuation compensation algorithm^40^. This procedure helped delineate dermis from epidermis (epidermis looks brighter) and the lymphatic vessels (dark areas in dermis) from the surrounding tissue. The dermis was then separated from epidermis through automatic segmentation^41^. As a next step, the compensated and segmented volumetric data was sliced in en face cross-sections and then the contrast of these slices was further improved using the histogram equalization algorithm^42^. Finally, the lymphatic vessels (dark areas) in the volumetric data were mapped using sorted minimum intensity projection (sMIP) technique. Please see Supplementary Data for details.

## Additional Information

**How to cite this article**: Baran, U. *et al.* OCT-based label-free *in vivo* lymphangiography within human skin and areola. *Sci. Rep.* **6**, 21122; doi: 10.1038/srep21122 (2016).

## Supplementary Material

Supplementary Information
