# Supplementary material for: OCT-based label-free in vivo lymphangiography within human skin and areola
Source: Sci Rep. 2016 Feb 19;6:21122. doi: 10.1038/srep21122 (PMC4759696; doi:10.1038/srep21122)
Supplement: Supplementary Information [file srep21122-s1.pdf]

OCT-based label-free in vivo lymphangiography within human skin and areola

Supplementary Information

Utku Baran[1,2], Wan Qin[1], Xiaoli Qi[1], Goknur Kalkan[3], and Ruikang K. Wang[1,*]

[1]Department of Bioengineering, University of Washington, Seattle, WA, USA;
[2]Department of Electrical Engineering, University of Washington, Seattle, WA, USA
[3]Department of Dermatology, Yıldırım Beyazıt University, Ankara, Turkey
[*]wangrk@uw.edu

Supplementary Figure 1 | Protocol for extracting lymphatic vessels within human dermis from volumetric OCT data

The figure shows the proposed protocol and an example result of a lymphatic network map. Here, the contrast of the original OCT structural images in coronal plane (i.e. B-scan) are first improved using attenuation compensation algorithm[1, 2], in step 1. This procedure helps delineate dermis from epidermis (epidermis looks brighter) and the lymphatic vessels (dark areas in dermis) from the surrounding tissue. The dermis is then separated from epidermis through automatic segmentation[3]. As a next enhancement step, the compensated and segmented volumetric data is sliced in enface cross-sections in transverse plane and then the contrast of these slices is further improved using the histogram equalization algorithm[4], in step 2. Finally, the lymphatic vessels (dark areas) in the volumetric data are mapped using sorted minimum intensity projection (sMIP) technique, where pixels with lower intensities are selected for projection map after sorting. Here, white color is used to map lymphatic vessels. Please see Supplementary Methods for details.

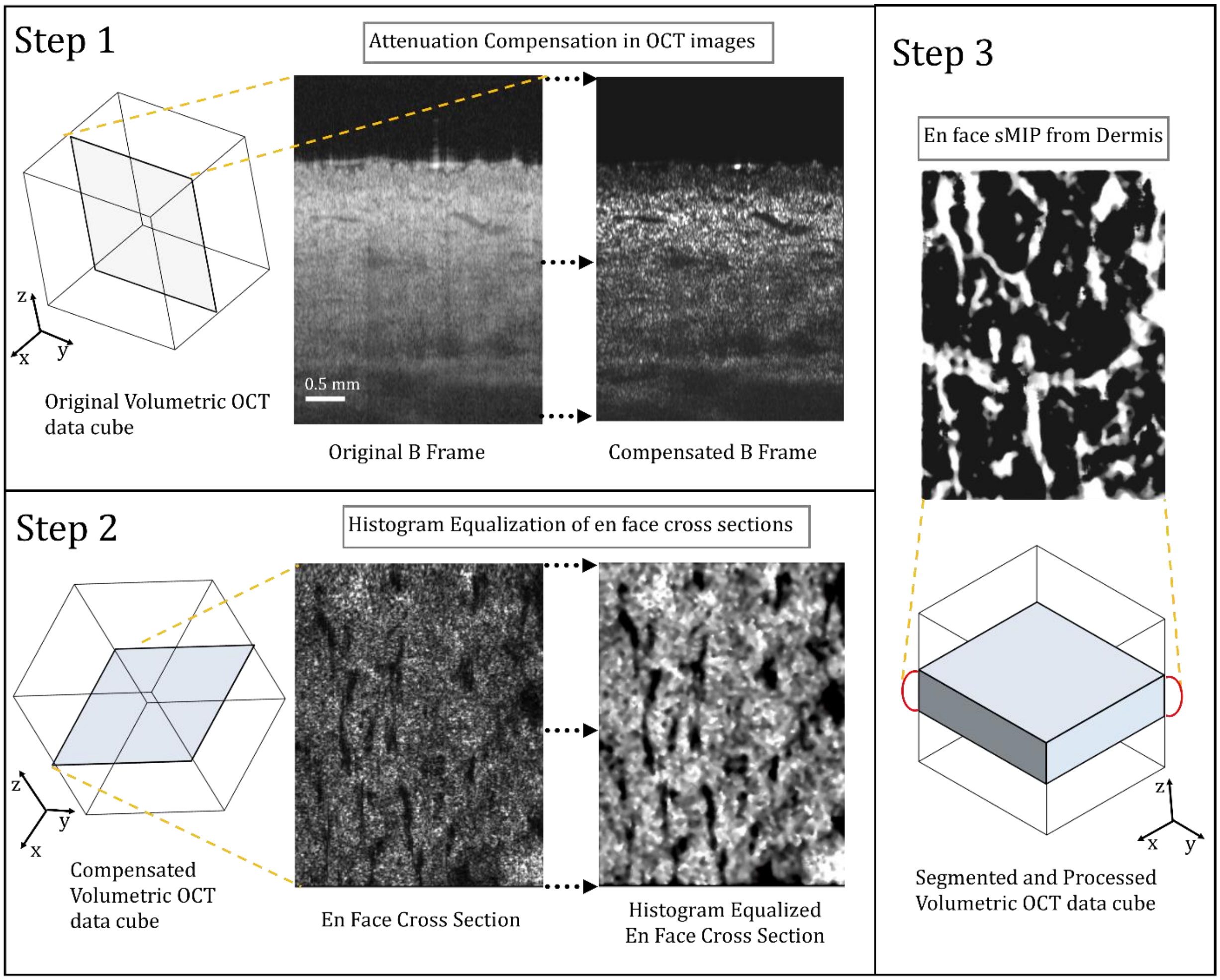

Supplementary Figure 2 | Swept-source OCT system

Portable bench top Swept-source OCT (SS-OCT) system with an MEMS-tunable vertical cavity surface-emitting laser (VCSEL) source engine is used. This VCSEL source (Thorlabs Inc.) is able to sweep the wavelength of the laser across a broad spectral range near 1310 nm at a fixed repetition rate of 100 kHz, giving a long coherence length (>50 mm) with ~12 µm axial resolution in tissue and 2 mm image range in human skin. The spectral bandwidth of laser output was measured ~67 nm at -3 dB. A Mach–Zehnder interferometer (MZI) built in the laser module provided real-time optical clocking, from which the MZI signal was detected by a dual-balanced detector connected to an external clock input of a 12 bit 500 MS/s data acquisition (DAQ) card. The measured sensitivity was 105 dB, almost constant up to depth of 4.25 mm in air. The sample arm consists of a probe imaging head (including galvanometric scanners) and a 5X objective lens (LSM03, Thorlabs Inc.), giving 22 µm lateral resolution.

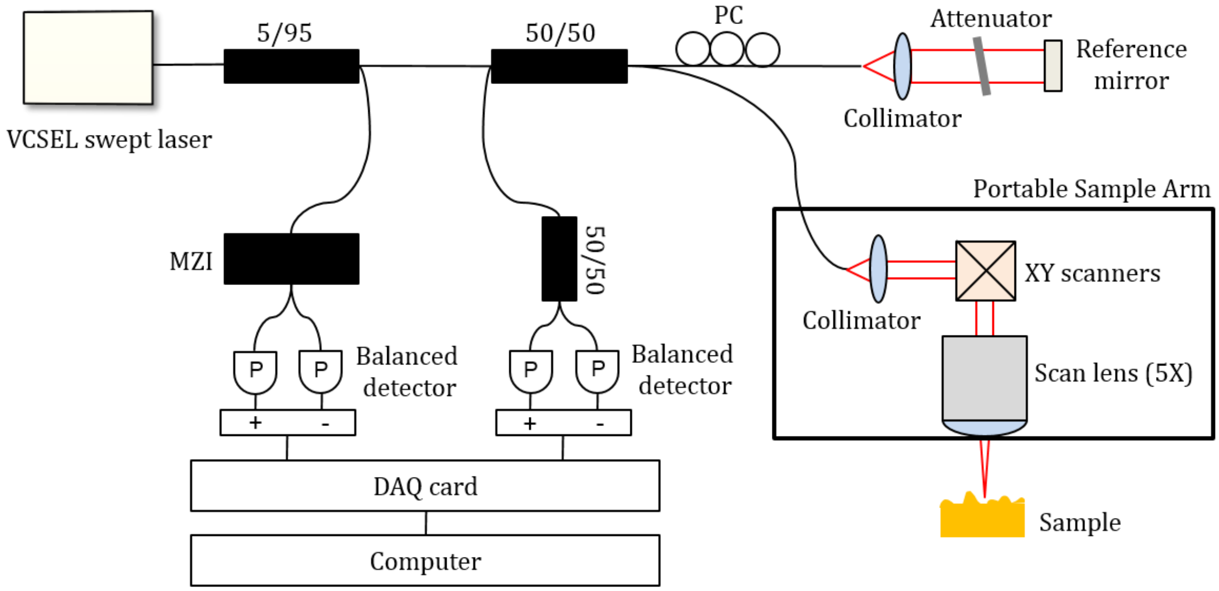

MZI: Mach–Zehnder interferometer; P: Photo-detector; DAQ card: Data acquisition card; PC: Polarization controller

Supplementary Figure 3 | Mouse ear imaging using proposed OCT methods

Imaging results of simultaneous optical microangiography and lymphangiography in the mouse ear after 5-minute depilation. Fig. 3a shows enface maximum projection view of the 3D OMAG image, Fig. 3b show sMIP of the 3D OCT lymphangiography. Fig. 3c is the overlay of Fig. 3a-b. Fig. 3d-f are the cross-sectional views of corresponding data sets from a location delineated with the orange dashed line. Fig. 3d is the OMAG image, Fig. 3e is the OCT structure image where lymphatic vessels are shown with green dashed circles, and Fig. 3f is the overlay of Fig 3d-e. The lymphangiography image acquired using our proposed method matches with the earlier reported results in literature[5, 6], which are validated using blue dye injection. The field of view of images is 2 × 2 mm^2.

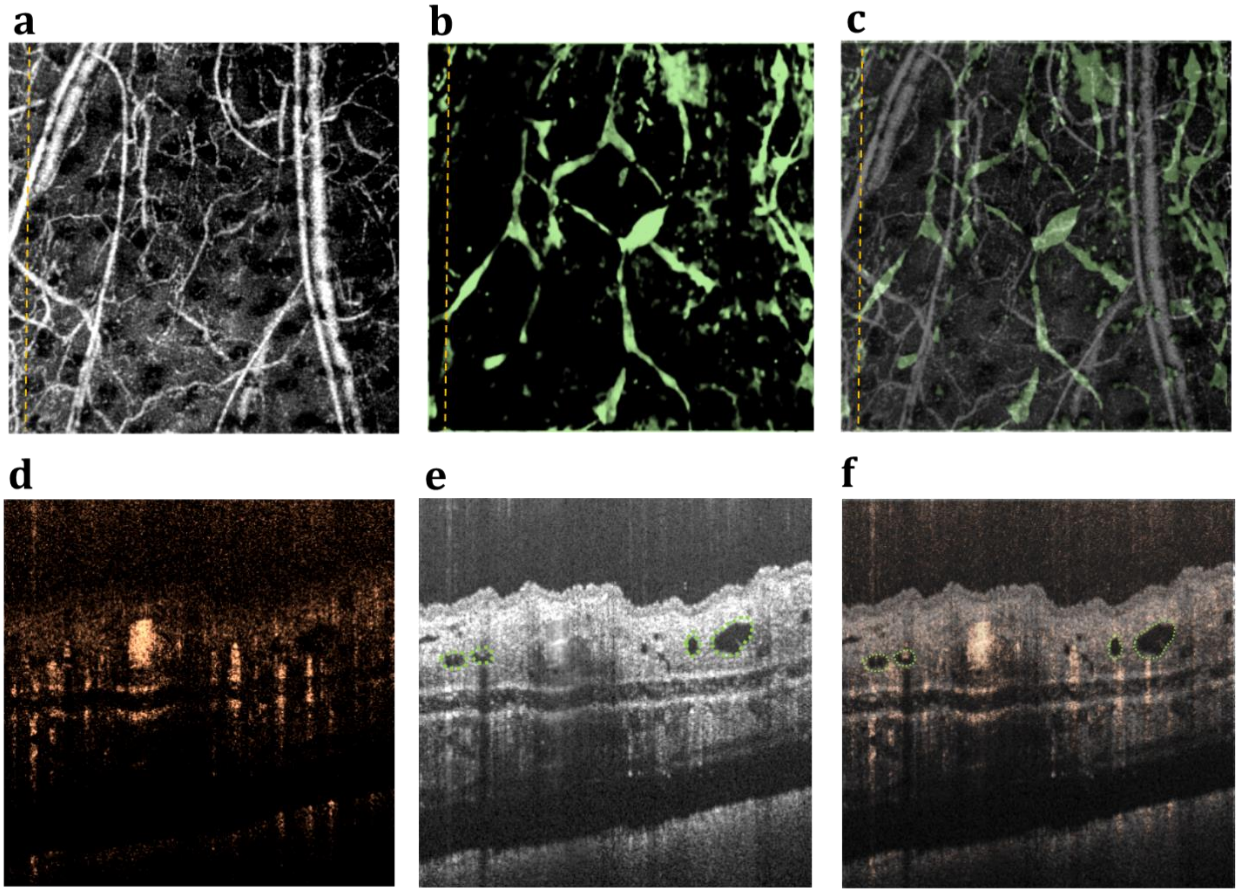

Supplementary Methods for OCT based Lymphangiography

Accurate detection of lymphatic vessels within human skin requires OCT images with good contrast. Non-uniform intensity distribution of the OCT images due to light attenuation and heterogeneous tissue properties make it difficult to extract the lymphatic vessels from the surrounding tissue with high scattering. In order to mitigate this problem, OCT structural images are processed in axial (A-scan or B-scan based processing) and lateral directions (enface based processing) and the contrast is enhanced using the algorithms described below. Please see Supplementary Fig. 1 for the proposed processing protocol.

Attenuation compensation in A-scan

In the standard SS-OCT system, the output *I* of the interference between a light beam reflected from a reference mirror and that from a sample can be described by Choma et al.[7]

$$I[k_m] = \frac{1}{2}\rho S[k_m](R_R + R_S + 2\sqrt{R_R R_S}\cos(2k_m \Delta x)) \quad (1)$$

where, k is the optical wavenumber, ρ is the detector responsivity, $S[k_m]$ is the source spectral density, Δx is the pathlength difference between the reference and the sample arms, and R_R and R_S are the reflectivity of the reference and the sample arm, respectively. The acquired signal ideally has values at M evenly spaced wavenumbers, so k= {k_1,k_2,..., k_m, ..., k_M}. The cross correlation term $H[k_m]$ is derived after removing the constant offset terms from Eq. 1:

$$H[k_m] = \frac{1}{2}\rho S[k_m]\sqrt{R_R R_S}\cos(2k_m \Delta x)) \quad (2)$$

Through a discrete inverse Fourier transform of Eq. 2, OCT A-scan, D, as a function of depth, z can be obtained:

$$D[z] = \sum_{k=1}^{M} H[k_m] e^{-2k_m z} \quad (3)$$

However, when light penetrates the tissue, it attenuates through the absorption (μ_a) and scattering (μ_s). This causes change in the acquired OCT A-scan signal by modifying Eq. 3 in its continuous form:

$$D_A(z) = D(z) e^{-2\alpha \int_0^z D(u)du} \quad (4)$$

where α is the backscattering coefficient, and D(μ) = $\mu_a + \mu_s$. When D(μ) is large at a certain depth, such as at a blood vessel, it shadows the remaining depth in that A-scan, D_A. This effect contributes to the poor contrast between the tissue and the lymphatic vessels in the deeper sections. To compensate for the attenuation in the OCT images, this decay term can be removed by using the following operation[2, 8]:

$$\int_{z=d}^{\infty} D_A(z) = \int_{z=d}^{\infty} D(z) e^{-2\alpha \int_0^z D(u)du} = \frac{e^{-2\alpha \int_0^z D(u)du}}{2\alpha}$$

$$\rightarrow e^{-2\alpha \int_0^z D(u)du} = 2\alpha \int_{z=d}^{\infty} D_A(z) \quad (5)$$

substituting Eq. 5 into Eq. 4 and discretising:

$$D[z] = \frac{D_A[z]}{2\alpha \sum_{z=d}^{\infty} D_A[z]} \approx \frac{D_A[z]}{2\alpha \sum_{z=d}^{F} D_A[z]} \quad (6)$$

The backscattering coefficient α can be adjusted depending upon tissue characteristics, and F is the final depth in the A-scan over which most of the light is attenuated. Note that this method does not require extracting attenuation coefficients μ_a and μ_s. Nevertheless, D[z] in Eq. 6 is the compensated reflection that allows the display of uniform images. This method assumes that the most of the light is attenuated within the recorded imaging depth range, and the backscattering coefficient is constant.

Histogram equalization of the en face cross sections

Due to the non-uniform mineral oil distribution topically applied onto the skin and the non-flat surface of the skin, the OCT image intensity is also non-uniform in enface cross sections. This situation is more severe when a large field of view is used such as in our case. Hence, additional OCT image enhancement step is needed before mapping the lymphatic vessels.

Histogram of an image is the probability distribution function of the occurrence of one specific grey level in that image. Histogram equalization is a contrast enhancement technique that increases the global contrast of images when the information content is represented by close contrast values. Histogram equalization method maps input intensities into an output image where its probability distribution function is uniform and its cumulative distribution function histogram is the same as the input image. This allows for stretching the contrast of the image to be distributed uniformly in the dynamic range[9]. However, it may produce unrealistic images and amplify background noise whilst decreasing the usable data. Histogram equalization has been generalized to local histogram equalization which divides the input image into smaller regions and applies histogram equalization algorithm to create a uniform distribution in each region[10]. Unfortunately, it creates noise amplification in homogenous regions.

To solve this problem, high peaks in the histograms, which correspond to homogenous regions in the image, are clipped and probability distribution function is non-uniformly reconstituted with Rayleigh distribution to enhance the foreground and supress the noise using equation below[4].

$$y = y_{min} + \sqrt{2\beta^2 \ln(\frac{1}{1-P_{input}(x)})} \quad (7)$$

Here, y is the output intensity, x is the input intensity, y_{min} is the low bound, *β* is the Rayleigh parameter and $P_{input}(x)$ is the cumulative probability of the input image. A higher *β* value will result in more contrast enhancement in the image while at the same time increasing saturation and noise levels. This technique transforms non-uniform, low-contrast OCT images into a uniform, high-contrast images which become useful in the mapping of the dark areas (supposedly lymphatic vessels) in the OCT images to a final enface image.

Enface mapping of lymphatic vessels

In order to segment the dermis region in the OCT images, we first detect the first layer of epidermis by extracting the first peak of each A-line. Then, we skip the epidermal layer starting from this first peak until the dermal-epidermal junction is reached by using a pre-determined number of pixels. Finally, we save the pre-determined pixels correspond to the papillary and reticular dermal layers in each A-scan. Repeating this procedure for every A-line flattens the curvature in the initial volume, and the interested volume can be automatically segmented with accuracy without the use of an additional treatment.

After segmenting dermis, we sort the pixels in each A-scan in ascending order, based on their intensities. Then we average the first N number of pixels with minimum intensities before mapping on to an enface 2D plane. In summary, Z_{en_face} [x, y] is calculated as:

$$Z_{en\_face}[x, y] = \frac{\sum_{i=1}^{M} Z_{sorted}[x,y,i]}{M} \quad (8)$$

Here, Z_{sorted}[x,y,i] is the value of the ith pixel in ascending order and M is the number of averaged pixels. We call this method sorted minimum intensity projection (sMIP). Final enface image is produced in inverted colors. The above procedures are illustrated in supplementary Fig. 1.

Supplementary References

1. Baran, U., Li, Y., & Wang, R. K. In vivo tissue injury mapping using optical coherence tomography based methods. *Applied optics* **54**, 6448-6453 (2015).
2. Vermeer, K. A., Mo, J., Weda, J. J. A., Lemij, H. G., & de Boer, J. F. Depth-resolved model-based reconstruction of attenuation coefficients in optical coherence tomography. *Biomedical optics express* **5**, 322-337 (2014).
3. Yin, X., Chao, J. R., & Wang, R. K. User-guided segmentation for volumetric retinal optical coherence tomography images. *Journal of biomedical optics* **19**, 086020 (2014).
4. Yousefi, S., Qin, J., Zhi, Z., & Wang, R. K. Uniform enhancement of optical micro-angiography images using Rayleigh contrast-limited adaptive histogram equalization. *Quantitative imaging in medicine and surgery* **3**, 5 (2013).
5. Vakoc, B. J. et al. Three-dimensional microscopy of the tumor microenvironment in vivo using optical frequency domain imaging. *Nature medicine* **15**, 1219-1223 (2009).
6. Yousefi, S., Zhi, Z., & Wang, R. K. Label-free optical imaging of lymphatic vessels within tissue beds in vivo. *Selected Topics in Quantum Electronics, IEEE Journal of* **20**, 15-24 (2014).
7. Choma, M., Sarunic, M., Yang, C., & Izatt, J. Sensitivity advantage of swept source and Fourier domain optical coherence tomography. *Optics Express* **11**, 2183-2189 (2003).
8. Girard, M. J., Strouthidis, N. G., Ethier, C. R., & Mari, J. M. Shadow removal and contrast enhancement in optical coherence tomography images of the human optic nerve head. *Investigative ophthalmology & visual science*, **52**, 7738-7748 (2011).
9. Gonzalez, R. C. *Digital image processing*. Pearson Education India. (2009).
10. Pizer, S. M. et al. Adaptive histogram equalization and its variations. *Computer vision, graphics, and image processing* **39**, 355-368 (1987).
